# Prophylactic zinc and therapeutic selenium administration in adult rats prevents long-term cognitive and behavioral sequelae by a transient ischemic attack

**DOI:** 10.1016/j.heliyon.2024.e30017

**Published:** 2024-04-25

**Authors:** Constantino Tomas-Sanchez, Victor Manuel Blanco-Alvarez, Juan Antonio Gonzalez-Barrios, Daniel Martinez-Fong, Guadalupe Soto-Rodriguez, Eduardo Brambila, Alejandro Gonzalez-Vazquez, Ana Karina Aguilar-Peralta, Daniel I. Limón, Viridiana Vargas-Castro, Jorge Cebada, Victorino Alatriste-Bueno, Bertha Alicia Leon-Chavez

**Affiliations:** a Facultad de Ciencias Químicas, Benemérita Universidad Autónoma de Puebla, 14 sur y Av. San Claudio, 72570, Puebla, Mexico; b Facultad de Enfermería, Benemérita Universidad Autónoma de Puebla, Av 25 Pte 1304, Colonia Volcanes, Puebla, Mexico; c Laboratorio de Medicina Genómica, Hospital regional 1° de Octubre, ISSSTE, Avenida Instituto Politécnico Nacional #1669, 07760, México D. F., Mexico; d Departamento de Fisiología, Biofísica y Neurociencias, Centro de Investigación y de Estudios Avanzados del Instituto Politécnico Nacional, Apartado Postal 14-740, 07000, México D.F., Mexico; e Nanoparticle Therapy Institute, 404 Avenida Monte Blanco, Aguascalientes, 20120, Mexico; f Facultad de Medicina, Benemérita Universidad Autónoma de Puebla, 13 Sur 2702, Col. Volcanes, 72410, Puebla, Mexico

**Keywords:** Growth factors, Nitrosative stress, Depression-anxiety, Reactive astrogliosis, Zinc, Selenium

## Abstract

The transient hypoxic-ischemic attack, also known as a minor stroke, can result in long-term neurological issues such as memory loss, depression, and anxiety due to an increase in nitrosative stress. The individual or combined administration of chronic prophylactic zinc and therapeutic selenium is known to reduce nitrosative stress in the first seven days post-reperfusion and, due to an antioxidant effect, prevent cell death. Besides, zinc or selenium, individually administered, also causes antidepressant and anxiolytic effects. Therefore, this work evaluated whether combining zinc and selenium could prevent stroke-elicited cognition and behavior deficits after 30 days post-reperfusion. Accordingly, we assessed the expression of growth factors at 7 days post-reperfusion, a four-time course of memory (from 7 to 28 days post-learning test), and cell proliferation, depression, and anxiety-like behavior at 30 days post-reperfusion. Male Wistar rats with a weight between 190 and 240 g) were treated with chronic prophylactic zinc administration with a concentration of 0.2 mg/kg for 15 days before common carotid artery occlusion (10 min) and then with therapeutic selenium (6 μg/kg) for 7 days post-reperfusion. Compared with individual administrations, the administration combined of prophylactic zinc and therapeutic selenium decreased astrogliosis, increased growth factor expression, and improved cell proliferation and survival in two regions, the hippocampus, and cerebral cortex. These effects prevented memory loss, depression, and anxiety-like behaviors. In conclusion, these results demonstrate that the prophylactic zinc administration combined with therapeutic selenium can reduce the long-term sequelae caused by the transient ischemic attack.

Significance statement.

A minor stroke caused by a transient ischemic attack can result in psychomotor sequelae that affect not only the living conditions of patients and their families but also the economy. The incidence of these micro-events among young people has increased in the world. Nonetheless, there is no deep understanding of how this population group responds to regular treatments (Ekker and et al., 2018) [1]. On the basis that zinc and selenium have antioxidant, anti-inflammatory, and regenerative properties in stroke animal models, our work explored whether the chronic combined administration of prophylactic zinc and therapeutic selenium could prevent neurological sequelae in the long term in a stroke rat model of unilateral common carotid artery occlusion (CCAO) by 10-min. Our results showed that this combined treatment provided a long-term neuroprotective effect by decreasing astrogliosis, memory loss, anxiety, and depression-like behavior.

## 1 Introduction

The transient ischemic attack (TIA) appears when the blood supply is briefly interrupted to the brain (from 10 to more than 60 min) [2]. TIA caused by a transient carotid artery occlusion is commonly known as a minor stroke or mini-stroke [3]. Its symptoms are weakness, paralysis, garbled speech, or understanding deficits [1]. Even when these symptoms are not permanent, long-term cognitive, motor, and sensory sequelae appear [[4], [5], [6], [7]], and depression, apathy, or anxiety have also been detected in one-third of stroke survivors [5,[8], [9], [10]]. Previous studies reported an increased frequency of ischemia in young people, and there is no effective treatment to date [[11], [12], [13]]. These studies have also shown that there is a relationship between sex, age, and ischemic stroke risk. While the risk is higher in middle-aged men, women have a minor risk in the early stages of life, but it increases with menopause and aging [14].

Depression, the most concerning sequela, is known to be triggered by several mechanisms, including alterations in the monoamine levels, decreased brain-derived neurotrophic factor (BDNF) levels [15], neuroinflammation, redox imbalance, and alteration in the hypothalamic-pituitary-adrenal axis [16,17]. In addition, the depression that occurs after a stroke has been proven to impede neuroplasticity [18] because, in general, stroke affects neurogenesis [19] and causes neuronal death [20], mainly in brain regions such as the frontal cortex, hippocampus, and hypothalamus [21].

Ischemia-reperfusion injury is known to reduce the general antioxidant activity by decreasing the expression of antioxidant enzymes such as superoxide dismutase (SOD), glutathione peroxidase (GPx), and glutathione redox ratio [[23], [24], [25]]. This loss of antioxidant defense is still exacerbated by the deficit of selenium (Se) [22] and zinc (Zn) [23]. In addition, the deficit of these micronutrients also decreases the expression of BDNF [26] and iodothyronine deiodinases (DIOs) [27,28], which correlates with depression and cognitive impairments (such as learning and memory) in both human and experimental animal models [[29], [30], [31]]. On the contrary, Zn administration prevents those ischemic impairments in the rat [[32], [33], [34]] because of its antidepressant and anxiolytic effects [[35], [36], [37]]. It is worth noting that systemic Se administration has been reported to increase GPx4 expression [38], reduce oxidative stress [39], promote mitochondrial biogenesis, and enhance the bioenergetic system. All these factors have been linked to enhanced cell survival in the penumbra zone [40] and prevention of cell death after a hemorrhagic or ischemic stroke [38]. However, there is insufficient research on the combination of Zn and Se. While previous studies have demonstrated decreased tissue damage during the early phase [34], the long-term neuroprotective remains unstudied.

Zn or Se are known to increase growth factor levels such as BDNF, nerve growth factor (NGF), and basic fibroblast growth factor (FGF2), after an ischemic process [33,41]. Among these factors, BDNF is differentiated by its role in memory functions, neurogenesis, and structural maintenance; its deficit has been associated with depression and neurodegeneration [42]. Accordingly, low peripheral BDNF levels match with cognitive impairment in stroke patients [43], and BDNF epigenetic alterations correlate with memory loss and depression [44]. FGF-2 also has antidepressant effects [45] and acts in response to stress [46]. NGF is involved in the growth development, survival of neurons, maintenance, and proliferation [47,48], but its role in depression is still controversial [49]. It has also been found that insulin-like growth factor 1 (IGF-1) levels are elevated in the serum of patients with major depression [50].

Previous studies have shown that Zn preconditioning administration and therapeutic Se mitigates inflammation by decreasing lipid peroxidation [34,54] and promoting BDNF expression after ischemia [55]. In addition, that dual treatment displays an antioxidant effect by inhibiting the NOS enzyme, increasing manganese SOD, copper/zinc SOD, and GPx4. Furthermore, the treatment also prevents excitotoxicity by inhibiting the NMDA receptor [[51], [52], [53]]. Our research group and other authors have demonstrated that the prophylactic subacute (4 days) [33] and chronic Zn administration (14 days) by intraperitoneal via [56], in the presence or absence of therapeutic Se, neuroprotects from a hypoxia-ischemia insult of 10 min or 30 min of common carotid artery occlusion (CCAO) at day 7 post-reperfusion in the rat [[32], [33], [34]]. Other animal models for transient ischemic attack are not frequently utilized in research; however, some studies have shown that a minor stroke in young people can have long-term sequels that become apparent in adulthood. Therefore, it is crucial to investigate the effects of combining prophylactic Zn and therapeutic Se treatment on cell survival and clarify the role of growth factors, which may account for the prevention of cognitive damage and astrogliosis induced by transient ischemia.

This work aimed to determine if the combined administration of chronic prophylactic Zn with therapeutic Se could maintain the neuroprotective effect 1-month post-reperfusion. We evaluated the expression of growth factors that have been proven to prevent the learning-memory deficit and confirmed these observations in the Morris water maze test. We also assessed depression/anxiety-like with the elevated plus maze and open field tests, the reactive astrogliosis and cell proliferation with immunohistochemistry assays, and nitrosative stress evaluated through nitrite and lipid peroxidation levels.

## 2 Methods

### 2.1 Experimental design

UPEAL-CINVESTAV supplied a total of 162 male Wistar rats of 2.5-month-old (body weight 190 g–240 g, RGD Cat# 13508588, RRID: RGD_13508588), which were housed in optimum room conditions with regulated temperature (22 ± 3 °C) and 12 h cycles of light-dark (light starting at 07:00 h). Groups with *n* = 3–6 rats in each study were kept in acrylic cages (34 cm × 44 cm X 20 cm). Food (Laboratory Autoclavable Rodent Diet 5010), containing 130 ppm of Zn and 0.47 ppm of Se (LabDiet; Saint Louis, MO, USA. Cat# 0001326) and drinking water *ad libitum*. The experimental procedures were authorized by the Institutional Animal Care and Use Committee under protocols 0162-15 CINVESTAV and 360-BUAP. These protocols comply with the current Mexican legislation NOM-062-ZOO-1999 (SAGARPA) and are based on the National Research Council's Guide for the Care and Use of Laboratory Animals (2010). Every effort was made to minimize animal suffering during the experiments [57].

#### 2.1.1 Unilateral transient common carotid artery occlusion (CCAO)

Surgeries were carried out on day 15 of the Zn administration period under strict aseptic conditions of surgical tools and the operating room. The animals were sedated with a mix of ketamine to 70 mg/kg and xylazine to 6 mg/kg at a dose of 200 μL/100 g of body weight intraperitoneally. First, we dissected the left common carotid artery through a 0.5 cm-long skin incision in the neck and then clamped for 10 min (Bulldog Clamps, INS6000119; Kent Scientific Corporation; Torrington, CT, USA). After the occlusion was finished, the arterial reperfusion was visually verified and the incision was sutured using 3-0 silk thread (Atramat; Ciudad de Mexico, Mexico). The animals were kept in individual cages while they recovered.

### 2.2 Zinc and selenium administration

The Zn preconditioning effect was achieved by daily injections intraperitoneal via of Zn chloride (ZnCl_2_, equivalent to 0.2 mg of Zn/Kg of body weight, in sterile water; Sigma-Aldrich; Saint Louis, MO, USA) for fourteen days before CCAO. The therapeutic Se was administrated via intraperitoneal, in the form of sodium selenite (Na_2_SeO_3_ in sterile water, equivalent to 6 μg of Se/Kg of corporal weight, (Sigma-Aldrich; Saint Louis, MO, USA) for 1 h before CCAO, and was continued every day for seven days (Days 15–21). These concentrations were reported in previous studies [34].

**Fig. 1:**
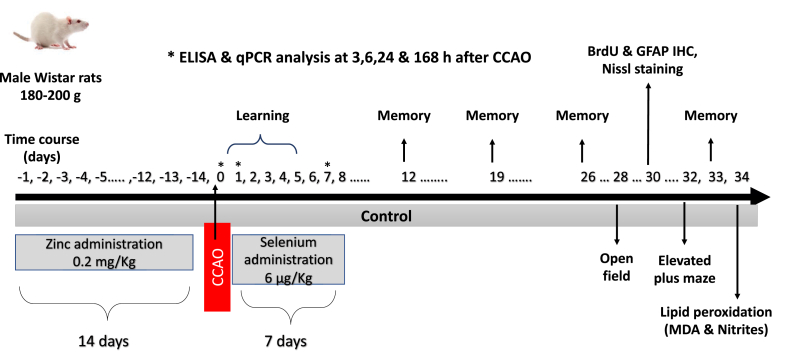
Schematic diagram of the experimental protocol.

Rats were grouped (*n* = 3–6) as follows: (1) **Control**, without treatment, and no surgery; (2) **Zn,** chronic zinc administration; (3) **Zn + Se**, chronic Zn administration and one Se administration on day 15; (4) **CCAO**, CCAO by 10 min; (5) **Zn + CCAO**, chronic Zn administration before CCAO; 6) **Zn + CCAO + Se**, chronic Zn administration before CCAO and therapeutic selenium treatment for seven days. Groups (1), (2), and (3) were euthanized on the treatment final day to obtain the brains (day 0). The brain tissue of groups (4), (5), and (6) was dissected at 3, 6, 24, and 168 h after reperfusion for qPCR assays. Groups (1), (2), (4), (5), and (6) were repeated with a new set of animals (n = 3–6) to evaluate histological assays, lipid peroxidation, and behavioral tests and were euthanized on day 34 post-reperfusion. All variables studied were assessed in two brain regions, the temporoparietal cortex and hippocampus, and each group was age-matched (see Fig. 1) [34].

### 2.3 Morris Water Maze

To determine if combined Zn and Se administration prevents long-term memory loss, we evaluated spatial reference memory with the Morris Water Maze (MWM). The experiments were performed on a rounded tank (150 cm diameter and 80 cm depth) filled with water and divided into four fictitious quadrants. A constant water temperature of 23 ± 2 °C was maintained. Multiple distant visual clues were painted on the walls of the MWM and the room. The evaluation consisted of five days of testing with four consecutive trials each day. Each animal was put in the tank facing the wall and permitted to swim unhindered to find an escape platform (15 cm in diameter and 40 cm high) submerged 2 cm under the water surface kept in the center of the southeast (S.E.) quadrant of the tank. In the first trial of test day, if the animals could not locate the platform within 60 s, they were gently led there, permitted to stay there for 30 s, and taken out of the tank. To evaluate the memory of the animals, the platform was kept in the same quadrant on days 7, 14, and 21 after the learning test. This was done to confirm that the animals remembered the visual-spatial details of the maze while swimming [34,58]. On day 28 following the learning test, long-term memory was assessed by removing the platform. We counted the times that rats passed the former platform position and their delayed arrival.

### 2.4 Open field

The open-field test is a method used to evaluate the level of locomotor activity, anxiety, and the presence of depressive-like behavior in rats.; this method has been used in several cerebral injury models [59]. All rats were evaluated on day 28 post-reperfusion. The wooden box used for evaluation was 60 cm on each side and 70 cm high. It was sectioned into numbered quadrants from 1 to 9. The duration of the test was 5 min, in which the following parameters were measured: time spent in each quadrant, the number of times the subject passed through the starting line (square # 1), the distance traveled (the result was multiplied by 20 cm), the vertical exploration (sum of the animal's standing-upright), the number of times the animal groomed itself and the number of fecal boluses and urination.

### 2.5 Elevated plus maze

Depressive and anxiety-like behavior was measured in all rats at day 32 post-reperfusion using the elevated plus-maze, due to the natural phobia of rats to open spaces. The labyrinth was elevated 90 cm above the floor and consisted of two enclosed arms (measuring 25 x 5 × 16 cm and painted black) and two open arms (measuring 25 x 5 × 0.5 cm) with a central platform (measuring 5 x 5 × 0.5 cm). The open arms had a 0.5 cm wall to prevent falls, while the closed arms had a 16 cm wall to provide complete coverage [60].

We conducted experiments for 5 min and recorded the number of times the animal visited the closed and open arms, the time spent in each arm, and the frequency of defecations and urinations.

### 2.6 Nitrites

To investigate the role of nitric oxide, the levels of nitrites were evaluated in the hippocampus and temporoparietal cerebral cortex at the end of the study. After 34 days of reperfusion, the hippocampus and temporoparietal cortex of five rats from each group were homogenized in a phosphate-buffered saline solution with a pH of 7.4. The homogenates were centrifuged at 12,500 rpm for 30 min at 4 °C using a Z 216 MK microcentrifuge (HERMLE Labortechnik, Wehingen, Germany). The absorbance of the samples was measured at a wavelength of 540 nm with a NanoDrop (Thermo Scientific Technologies; Wilmington, DE, USA), and the production of NO was calculated by interpolating values in a standard curve of NaNO_2_ (1–10 μM) in the supernatants, as follows: 10 μL of the supernatant plus 10 μL of Griess reagent composed of 0.1 % N-(1-naphthyl) ethylenediamine dihydrochloride and 1.32 % of sulfanilamide in 60 % acetic acid (1:1) to produce a colorimetric reaction. Complete details of this procedure are described in Ref. [61].

### 2.7 *Lipid* peroxidation

The nitric oxide reacts with the superoxide anion to produce peroxynitrite, which causes the oxidation of lipids and can be detected by the Gérard-Monnier method. Malondialdehyde (MDA) and 4-hydroxyalkenals (4-HDA) were measured at 34 days post-reperfusion in the same supernatant used for nitrite detection of (*n* = 5 rats per group) as described elsewhere [62]. To generate the colorimetric reaction, 200 μL of the supernatant was mixed with 650 μL of 10.3 mM N-methyl-2phenyl-indole (Sigma-Aldrich; Saint Louis, MO, USA), diluted in a mixture of acetonitrile and methanol in a 3:1 ratio. Further, 150 μL of methanesulfonic acid (Sigma-Aldrich; Saint Louis, MO, USA) was added to the mixture. After the reaction mixture was vortexed, it was incubated at 45 °C for 1 h. Following this, it was centrifuged at 3000 rpm for 10 min. The sample was then measured at 586 nm using a Smart-Spec 3000 spectrophotometer (Bio-Rad; Hercules, CA, USA). A standard curve was compared in the concentration range of 0.5–5 μM of 1,1,3,3-tetramethoxypropane (10 mM stock) to determine the content of MDA + 4-HDA in each sample.

### 2.8 Reverse transcription

Total ribonucleic acid (RNA) was obtained from Samples (100 mg per brain region) of the temporoparietal cortex and the hippocampus of experimental and control rats, adding 1 mL of TRIzol (Invitrogen Corporation; Carlsbad, CA, USA) and using a NanoDrop Spectrophotometer for its quantification (Thermo Scientific NanoDrop Technologies; Wilmington, DE, USA). To obtain cDNA, we used 5 μg of total RNA from three rats per group. We added 1 μL of SuperScript II reverse transcriptase (Catalog 18080093, Invitrogen; Carlsbad, CA, USA), 1 μL of Oligo dT 50 μM, 1 μL of dNTP mix 10 mM, and molecular biology grade water up to 13 μL. The reverse transcription process was carried out under the following conditions: denaturation at 70 °C for 10 min, followed by hybridization at 42 °C for 5 min cDNA synthesis was performed at 55 °C for 50 min, and then the mixture was heated to 70 °C for 15 min. Residual RNA was eliminated using 1 μL of RNase H (Invitrogen; Carlsbad, CA, USA) at 37 °C for 20 min.

### 2.9 qPCR

Each gene was amplified from the cDNA samples using TaqMan probes (Thermo Fisher Scientific; Waltham, MA, USA) (Table 1). In a final volume of 5 μL, the amplification reactions comprised 0.25 μL of the corresponding TaqMan probe, 2.5 μL of Master Mix (TaqMan Universal Master Mix by Life Technologies in Carlsbad, CA, USA), and 2.25 μL of cDNA sample. For qPCR, the settings were denaturation for 10 min at 95 °C, amplification for 45 cycles of 15 s each, and reaction end for 1 min at 60 °C. The PCR system used was a 7900HT Fast Real-Time PCR System (Applied Biosystems; Foster City, CA, USA). Rat β-actin was used as a housekeeping for the normalization of amplicon quantifications. The 2-ΔΔCt analysis is used to compare the gene expression levels between a treated group and a control group. The analysis is double normalized using first housekeeping genes, and second the control group without treatment.

**Table 1:** TaqMan probes for Real-time PCR assay.

Gene	Gene name	Essay
*Igf1*	Insulin-like growth factor 1	Rn00710306_m1
*Bdnf*	Brain-derived neurotrophic factor	Rn02531967_s1
*Vegf*	Vascular endothelial growth factor	Rn01511602_m1
*Gdnf*	Glia-derived neurotrophic factor	Rn00569510_m1
*Fgf2*	Fibroblast growth factor 2	Rn00570809_m1
*Ngf*	Nerve growth factor	Rn01533872_m1

### 2.10 Enzyme-linked immunosorbent assay (ELISA)

The ELISA technique was used to measure protein levels of BDNF, NGF, IGF-1, FGF2, and VEGF in the hippocampus and temporoparietal cortex homogenates (n = 5 rats per group). All the samples were processed simultaneously in the same ELISA plate to avoid external interference in the experiment. The temporoparietal cortex was homogenized in 600 μL of PBS, and the hippocampus in 300 μL of PBS. After protein quantification by the Sedmak and Grossberg method, 5 μg of protein was put into the respective wells of the ELISA plate. To begin the experiment, 100 μL of 0.1 M carbonate buffer was added to the plate, which was then incubated at 4 °C for 18 h. After the incubation period and washing, 200 μL of 0.5 % bovine serum albumin (IgG free) was added to each well. This was left for 30 min at room temperature (RT) to block non-specific binding sites. The wells were washed thrice with PBS–Tween 20 (0.1 %) solution. Then, primary antibodies were added to each well 100 μL (1:500 dilution) and incubated for 2 h at RT. They were against BDNF (Abcam Cat# ab203573, RRID:AB_2631315), NGF (Abcam Cat# ab6199, RRID:AB_2152414), IGF-1 (Abcam Cat# ab9572, RRID:AB_308724), and FGF2 (Abcam Cat# ab126861, RRID:AB_11131940). The wells were washed three times with PBS–Tween 20 (0.1 %). Then, 100 μL of a horseradish peroxidase-conjugated goat anti-rabbit or goat anti-mouse IgG (1:1000 dilution; Dako North America Inc; Carpinteria, CA, USA) was added to the wells and left to incubate at RT for 2 h. After that, the antibody-antigen complex was revealed by adding 100 μL of 2,2′-azino-bis (3-ethylbenzothiazoline-6-sulfonic acid) (ABTS) containing 0.3 % H_2_O_2_ to each well. Finally, after a 15-min duration, the optical density (OD) was measured at 415 nm with a Benchmark microplate reader (Bio-Rad; Hercules, CA, USA).

### 2.11 Proliferation assay by BrdU administration

We used the Bromodeoxyuridine (BrdU) protocol to investigate the long-term effects of Zn and Se administration on cellular proliferation induced by growth factors. BrdU (Sigma-Aldrich; Saint Louis, MO, USA) was dissolved at a concentration of 15 mg/mL in a sterile-filtered physiological saline solution (0.9 % NaCl) and intraperitoneally injected in each group of rats at a dose of 50 mg/kg of body weight for 4 consecutive days starting on the day of the CCAO. Each group was euthanized 30 days post-CCAO.

### 2.12 Immunohistochemistry for BrdU or GFAP

Immunohistochemistry against BrdU was used to evaluate cell proliferation and against glial fibrillary acid protein (GFAP) to assess astrogliosis in the hippocampus and the temporoparietal cortex. Sucrose-cryoprotected brains were sliced into 40 μm sagittal sections in all ipsilateral hemispheres to determine the number and distribution of BrdU or GFAP-positive cells. Free-floating tissue slices were washed thrice with TBS-1X for 5 min. Then, tissues were incubated with 3 % H_2_O_2_ for 30 min to block unspecific sites. For BrdU immunohistochemistry, the tissue samples were treated in a 2 N HCl solution at a temperature of 37 °C for 30 min. Then, the samples were neutralized using a 0.1 M borate buffer at room temperature for 12 min. Next, the tissues were blocked with TBS++ (which contains 10 % goat serum and 0.1 % Triton X 100) for 30 min. Following this, the samples were incubated with primary antibodies that are anti-BrdU (1:250 dilution; Bio-Rad Cat# MCA2483, RRID: AB_808349; Hercules, California, USA) or anti-GFAP (1:100 dilution; Cell Signaling Technology Cat# 3670, RRID: AB_561049, United States of America) for 48 h at 4 °C. After that, tissues were rinsed thrice with TBS-1X and were incubated with mouse peroxidase-labeled IgG and the VECTASTAIN® Elite ABC-HRP Kit for 4 h (Vector Laboratories Cat # PK-4002, RRID: AB_2336811); the immunohistochemistry was revealed with 3,3′ diaminobenzidine (DAB; Vector Laboratories). The immunostained tissues were carefully placed on gelatin-coated slides. Images of BrdU and GFAP-positive cells were taken by a bright field microscope coupled to a Progress Res C10 camera (Leica DM 1000 LED) using a 10X objective. The micrographs were merged using Adobe Photoshop CS6 to reconstruct the dentate gyrus and temporoparietal cortex from the ipsilateral hemisphere. The cells were then counted using ImageJ software (ImageJ, RRID: SCR_003070). [63].

### 2.13 Statistical analysis

The results presented are the mean ± SEM of 3–6 independent experiments, including controls. All values were normalized using Snedecor's F test and were compared to the untreated control, except for the qPCR values. The qPCR values were expressed as fold change (2^−ΔΔCt^). The results of behavioral tests, immunohistochemistry, and biochemistry parameters were analyzed with multiple-group comparison two-way ANOVA test and Dunnett, Tukey, or Bonferroni's posthoc when appropriate. Significance was *P* < 0.05 (*) when comparing each group vs. control or *P* < 0.05 (†) when comparing each group vs. CCAO. Statistical analyses were conducted using GraphPad Prism 10 software (RRID: SCR_0158070), which provides scientific analysis tools for quantitative and categorical data analysis as well as visualization and graphing capabilities.

## 3 Results

### 3.1 Zinc and selenium prevent memory loss long-term after reperfusion

**Fig. 2:**
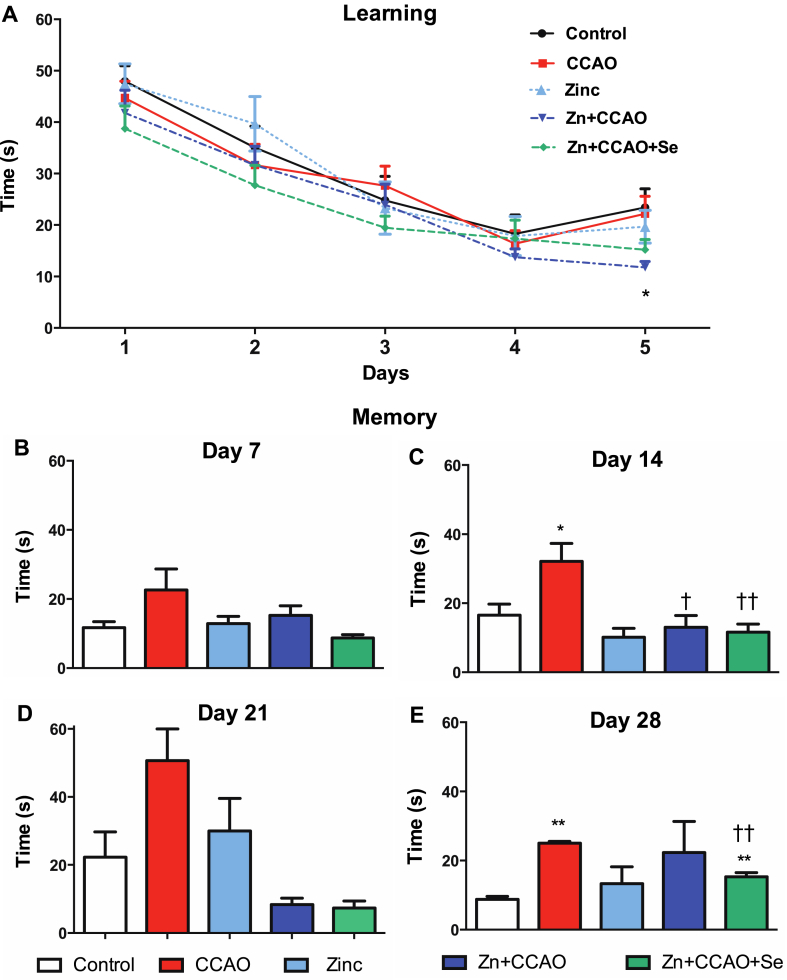
Prophylactic zinc and therapeutic selenium administrations improved spatial learning and memory. A) Five-day learning test in the Morris water maze. Memory was evaluated over days post-learning, as shown in the headings (B, C, D, and E). Values represent the mean ± SEM of 6 rats and their statistical differences were analyzed using a two-way ANOVA. Statistical significance was indicated as *P < 0.05, or **P < 0.01, when compared to the Control group, and †*P* < 0.05 and ††*P* < 0.01, vs. the CCAO group.

CCAO did not alter learning in a short-term period. The learning test in the MWM showed no difference between the CCAO group and the controls (*n* = 6) (Fig. 2A). However, the prophylactic administration of zinc before CCAO (Zn + CCAO) decreased the escape latency on day 5 (−35 ± 8.5 %, *P* < 0.05) of learning compared with the control group (* ANOVA, *P* < 0.05, post hoc Dunnett's test).

The study assessed long-term memory at various intervals, every 7 days until 28 days post-learning when the platform was retired. Tests performed at day 7 had no significant change (Fig. 2B). The CCAO group had a higher latency to find the escape platform on days 14 (194 ± 13.7 %, *P* < 0.01, Fig. 2C) and 28 (175 ± 3 %, *P* < 0.004, Fig. 2E) compared with the respectively with the control group. Zn + CCAO decreased the latency at 14 days post-learning. Only the combined treatment reduced by 34 ± 3 % (*P* < 0.002) the CCAO-induced rise on day 28 post-learning but with a higher latency than the control group (70 ± 5 %, *P* < 0.007, Fig. 2E). The results showed that the prophylactic administration of zinc maintained neuronal functionality for 14 days, and the combined treatment for 28 days after learning the escape task in the MWM test (Fig. 2D).

### 3.2 Zinc and selenium prevent depression/anxiety-like behavior long after reperfusion

**Fig. 3:**
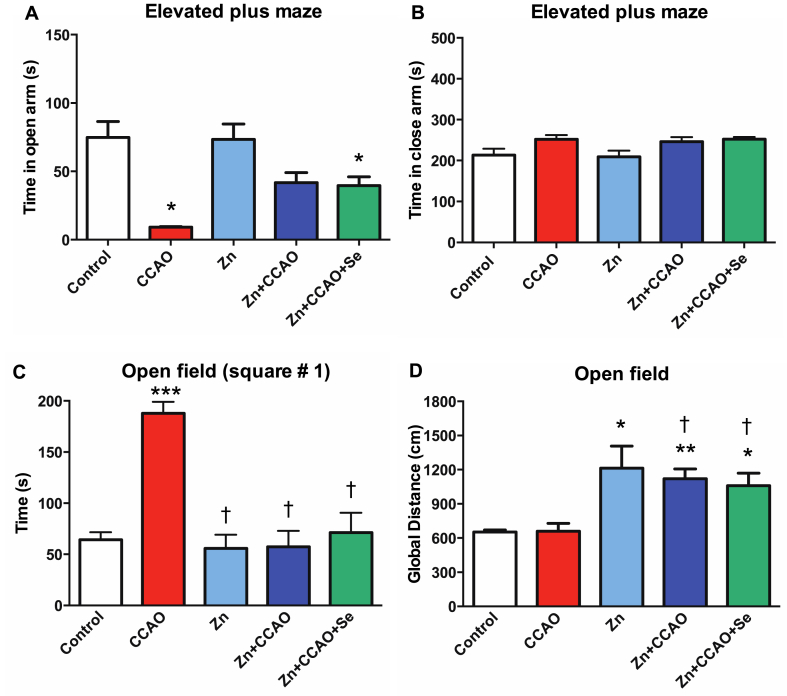
Prophylactic zinc and therapeutic selenium administrations prevented anxiety and depression-like behavior evaluated in elevated plus-maze (A and B) and recovered motor activity (C and D). Values represent the mean ± SEM of 6 rats. Statistical significance was indicated as *P < 0.05, **P < 0.01, and ***P < 0.001, Two-way ANOVA test and Tukey's multiple-group comparison analysis vs. the control group and †*P* < 0.05 when comparing with the CCAO group.

The CCAO group displayed depression/anxiety-like behavior in the elevated plus maze test compared to the control group, as indicated by the short permanence in the open arms (87 ± 1 %, P < 0.05, Fig. 3A). In contrast, the permanence duration was longer in the group treated with Zn + CCAO + Se compared to the group treated with only CCAO, thus suggesting that combined Zn and Se administration prevented the depression/anxiety-like behavior induced by CCAO. While in the enclosed arms, we did not observe any significant differences among all the groups (Fig. 3B).

The CCAO group showed a 192 ± 17 % increase in permanence at the starting line (square #1) compared to the control group in the open-field test (P < 0.05) (Fig. 3C), confirming that depression and anxiety-like behavior can develop after hypoxia-ischemia. However, CCAO did not alter the distance traveled during an open-field test compared to the control group (Fig. 3D). The prophylactic chronic Zn administration only or combined with Se avoided the CCAO-induced long permanence in the starting line (square # 1) (Fig. 3C). The treatments increased the distance traveled in the open field arena compared to the control and CCAO groups, including the chronic zinc administration alone (Zn group) (Fig. 3D). These results suggest that all therapeutic strategies tested can improve exploratory behavior.

### 3.3 Zinc and selenium prevent reactive astrogliosis long after reperfusion

**Fig. 4:**
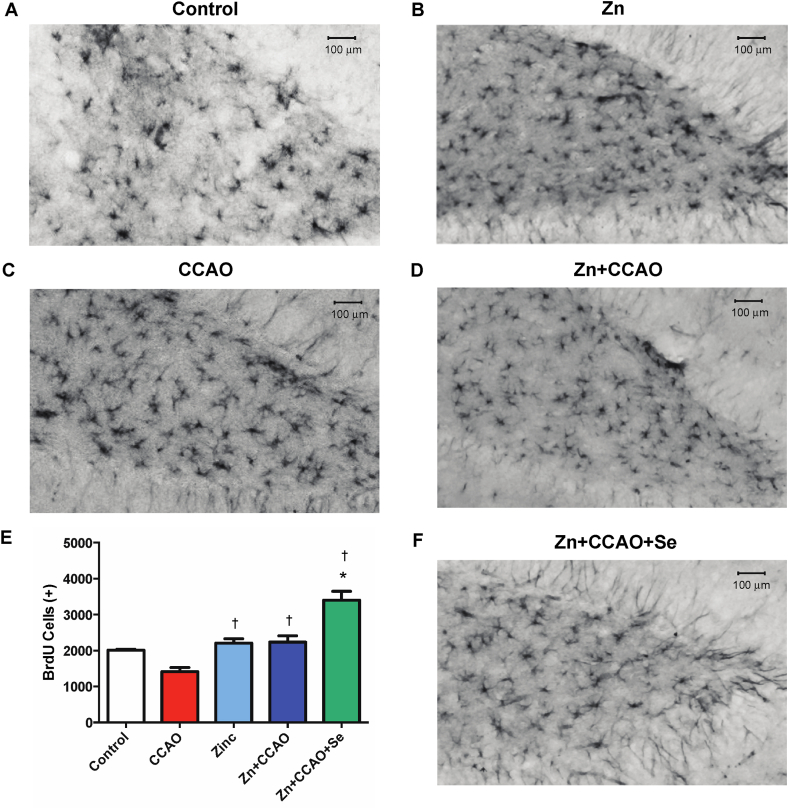
Combined prophylactic zinc and therapeutic selenium administration decreased astrogliosis in the hippocampus. The representative micrographs are integrated from several 20X amplifications of immunohistochemical staining. The optical density (OD) of GFAP is expressed on the graph; the background was subtracted from the measurement. The graph shows values expressed by the mean ± SEM of 24 slices per brain of 3 independent brains (Fig. 4E). The two-way ANOVA test with Bonferroni's correction was applied for multiple-group comparison against the control (*P < 0.05) and when compared with CCAO group (†*P* < 0.05).

**Fig. 5:**
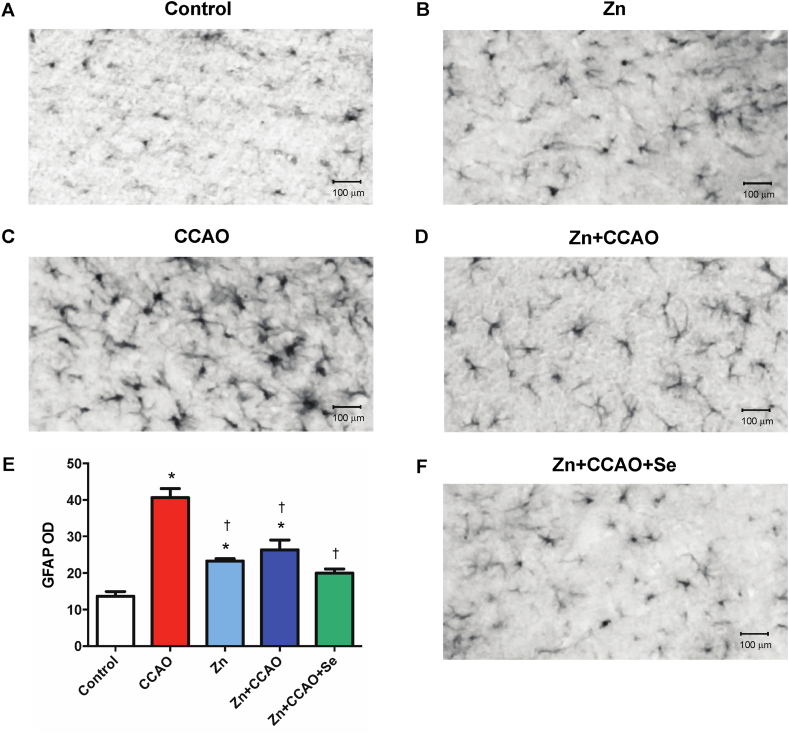
Prophylactic zinc and therapeutic selenium administrations decrease astrogliosis in the temporoparietal cortex. The representative micrographs are integrated from several 20X amplifications of immunohistochemical staining. The optical density (O.D.) of GFAP is expressed on the graph; the background was subtracted from the measurement. Values are the mean ± SEM of 24 slices of 3 independent brains. A two-way ANOVA test with Bonferroni's multiple-group comparison analysis. *P < 0.05 compared with the Control group and †*P* < 0.05 compared with the CCAO group.

Reactive astrogliosis was also evaluated in the late ischemic phase through GFAP immunoreactivity (Fig. 4, Fig. 5). The ischemic group exhibited more density of GFAP immunoreactivity than the control group (Fig. 4A and E), both in the hippocampus (133.7 ± 16.7 %, *P* = 0.01, Fig. 4C and E) and the temporoparietal cortex (197.7 ± 31.2 %, *P* = 0.01, Fig. 5C and E) compared with the control group (Fig. 5A and E). The prophylactic zinc administration alone (Zinc group) increases GFAP immunoreactivity in the temporoparietal cortex (70.3 ± 8.1 %, *P* < 0.05 Fig. 5B) but not in the hippocampus (Fig. 4B) compared with the control group. The prophylactic Zn administration (Zn + CCAO group) decreased GFAP immunoreactivity in the temporoparietal cortex (35.2 ± 11.3 %, *P* = 0.01, Fig. 5D and E) compared with the CCAO group. Interestingly, the Zn + CCAO + Se group also significantly decreased the CCAO-induced GFAP density in the hippocampus (27.4 ± 10.6 %, *P* = 0.01, Fig. 4F and E) and the temporoparietal cortex (50.9 ± 4.9 %, *P* = 0.01, Fig. 5F and E), supporting the neuroprotective effect of the combined treatment.

### 3.4 Zinc and selenium prevent lipid peroxidation long after reperfusion

**Fig. 6:**
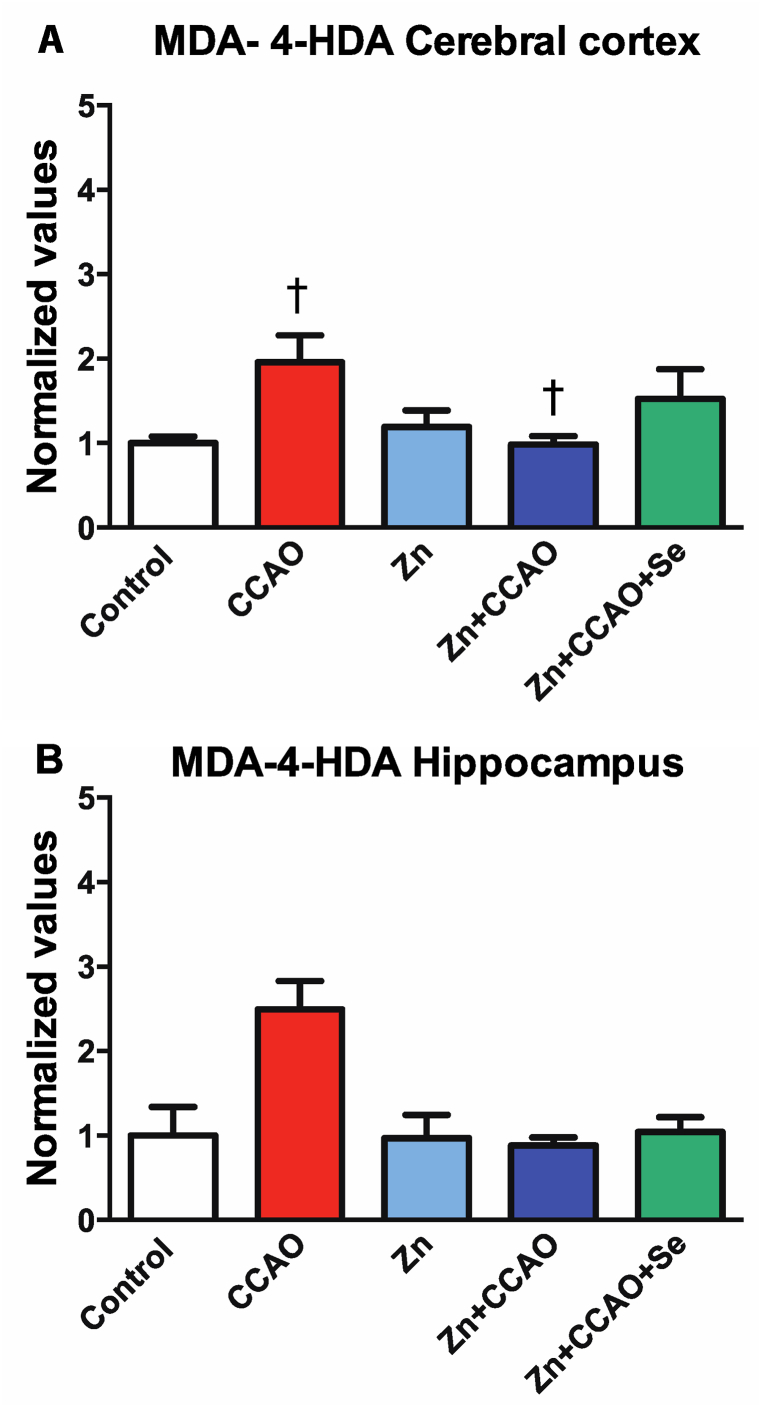
Prophylactic zinc and therapeutic selenium administrations prevented lipid peroxidation caused by ischemia in the temporoparietal cortex on day 34 post-reperfusion. Values represent the mean ± SEM of n = 6 rats. Statistical differences are indicated as *P < 0.05, two-way ANOVA with Dunnett's post hoc compared to the control group and †*P* < 0.05 with the CCAO group.

Nitrite levels in the temporoparietal cortex were similar across all groups on day 34 after reperfusion, which is the late phase of hypoxia-ischemia (data not shown). However, the ischemic group exhibited increased MDA and 4-had levels (95 ± 31 %, *P* = 0.04) in the temporoparietal cortex compared with the control group (Fig. 6A). Only the prophylactic zinc administration (Zn + CCAO) decreased by 11 ± 3 % the CCAO-induced increase in MDA and 4-HDA levels (*P* = 0.02; Fig. 6A). In the hippocampus, the experimental groups had no significant differences in either nitrites (data not shown) or lipid peroxidation (Fig. 6B).

### 3.5 Zinc and selenium increase the expression of growth factors in the temporoparietal cortex after reperfusion

We evaluated the expression of *Bdnf, Ngf*, *Fgf2, Vegf, Igf-1, and Gdnf* genes because of their involvement in the hypoxic-ischemic insult in the brain. In the hippocampus, mRNA and protein changes were not observed in all conditions except for *Fgf2* and *Bdnf* genes (data not shown). In contrast, all the genes studied were upregulated in the CCAO group in the temporoparietal cortex compared with basal mRNA levels used for normalization (values are shown in Table 2, *P < 0.05 when compared with the control group). Also, the treatments caused different effects on gene expression in this cerebral region depending on the time post-reperfusion.

**Table 2:** Gene expression of growth factors in the temporoparietal cortex.

	Hour	CCAO	Zn + CCAO	Zn + CCAO + Se
*Igf*-1	0	n.s	3.3 ± 0.9^b^	3.3 ± 0.9^b^
	3	6 ± 17^b^	3.2 ± 0.08^c^	9.2 ± 0.4^c^
	6	4.84 ± 1.8^b^	6.82 ± 0.2^c^	7.9 ± 2.2^c^
	24	0.51 ± 0.05^c^	2.88 ± 0.14^c^,	2.83 ± 1.2^c^,^d^
	168	3.3 ± 0.9^b^	2.96 ± 0.28^c^	5.2 ± 0.7^c^
*Fgf2*	0	n.s	3.5 ± 1.2^c^	8.6 ± 1.8^c^
	3	4.5 ± 1.5^b^	3.28 ± 0.5^c^	10.3 ± 0.7^c^,^d^
	6	n.s	6.23 ± 1^c^	4.9 ± 0.7^b^
	24	n.s	n.s	1.9 ± 0.2^c^,^d^
	168	2.69 ± 0.49^b^	3.11 ± 0.26^c^	3.62 ± 0.9^b^
*Bdnf*	0	n.s	9.63 ± 1.82^c^	23.8 ± 4.9^c^
	3	3.99 ± 1.1^c^	3.98 ± 1.4^b^	18.4 ± 2.7^c^,^d^
	6	5.93 ± 0.3^b^	17 ± 0.8^c^^d^	4.09 ± 2^a^
	24	0.68 ± 0.12^b^	n.s	5 ± 1^b^,^d^
	168	6.11 ± 2^b^	5.82 ± 0.57^c^	9.19 ± 0.4^c^
*Ngf*	0	n.s	15.4 ± 1^c^	20.95 ± 3.78^c^
	3	4.5 ± 0.62^c^	7.7 ± 0.4^c^,^d^	21 ± 1.12^c^,^d^
	6	7.5 ± 0.44****	15.3 ± 3.8^c^	12.12 ± 1.21^c^,^d^
	24	2.87 ± 0.37^b^	4.7 ± 0.85^c^,^d^	5.48 ± 1.47^c^
	168	3.43 ± 1.1^b^	10 ± 1.44^c^,^d^	10 ± 1.92^c^,^d^
*Vegf*	0	n.s	5.4 ± 0.3^c^	4.17 ± 0.54^b^
	3	n.s	4.69 ± 0.9^b^,^d^	5.21 ± 0.34^c^,^d^
	6	2.96 ± 0.6^b^	2.8 ± 0.55^b^	n.s
	24	n.s	4.13 ± 2.32^a^,^d^	n.s
	168	3.57 ± 0.08^c^	4.09 ± 0.33^c^	4.23 ± 0.44^a^
*Gdnf*	0	n.s	2.8 ± 0.9^b^	3.68 ± 1^b^
	3	n.s	n.s	n.s
	6	3.23 ± 0.92^b^	5.7 ± 1.29^c^	3.1 ± 0.45^c^
	24	n.s	n.s	n.s
	168	2.6 ± 0.7^b^	6.13 ± 1.07^c^,^d^	7.8 ± 1.27^c^,^d^

The fold change values of mRNA measured by qPCR Taqman are expressed as median ±SEM of *n* = 3 for each group and time studied. Two-way ANOVA analysis and Dunnett's post hoc test showed statistical differences of.

a *P* < 0.05.

b *P* < 0.01.

c *P* < 0.0001 compared with the control group without treatment and.

d *P* < 0.05, compared with the CCAO group; n.s, not significant v.s. the control group.

The results for the fold change in mRNA compared with the CCAO group are described in the text (shown as †*P* < 0.05, Table 2). The impact of the treatments was as follows: only the combined treatment (Zn + CCAO + Se) increased *Igf-*1 mRNAs in all times studied (Table 2). The Zn + CCAO + Se group showed a higher *Fgf2* transcriptional rate from 3 h (2.2 ± 0.1, *P* = 0.001) to 24 h (6.59 ± 0.4, *P* = 0.001). The prophylactic zinc administration alone or combined with therapeutic selenium upregulated *Bdnf* mRNA (Table 2). Even yet, the combined treatment values were statistically higher than the prophylactic zinc administration at 3 h (4.3 ± 0.13, *P* = 0.0005) and 24 h (7.4 ± 1.8, *P* = 0.001) post-reperfusion (Table 2). Conversely, in the hippocampus, *Bdnf* mRNA had an upregulation in the Zn + Se (2.2 ± 0.4, *P* = 0.05) and Zn + CCAO groups at 24 h (2.16 ± 0.32, *P* = 0.02). The transcriptional level of *Vegf* and *Gdnf* did not present significant changes by the treatments except at 24 h for *Vegf and Gdnf* in the Zn + CCAO group (2.35 ± 0.41, *P* = 0.01) and at 168 h in the Zn + CCAO + Se group reperfusion (3 ± 0.4, *P* = 0.001) (Table 2).

However, the protein values presented variable increments and groups in the cerebral cortex at some times. The increase in Igf-1 protein levels was only significant at 168 h post-reperfusion in the Zn-CCAO (73 ± 3 %, *P* = 0.001) and Zn + CCAO (94 ± 2 %, *P* = 0.001) groups. However, FGF2 protein levels increased in the groups of CCAO (59 ± 12 %, *P* = 0.003), Zn + CCAO (72 ± 14 %, *P* = 0.001) at 6 h post-reperfusion and Zn + CCAO + Se at 168 h (53 ± 5 %, *P* = 0.01) post-reperfusion. In the hippocampus, a significant increase in FGF2 protein levels (49 ± 4 %, *P* = 0.001) was observed in the Zn + CCAO group at 3 h post-reperfusion. However, BDNF protein expression statistically increased only in the temporoparietal cortex in Zn + CCAO (55 ± 23 %, *P* = 0.04) and Zn + CCAO + Se groups (41 ± 4 %, *P* = 0.002) at 24 h post-reperfusion. Although Ngf mRNA was upregulated only in the Zn + CCAO + Se group (Table 2), NGF protein levels only increased in the CCAO group at 3 h (142 ± 8 %, *P* = 0.0001) and 6 h (44 ± 1 %, *P* < 0.001) post-reperfusion. The protein levels of the other growth factors studies were unaltered.

### 3.6 Zinc and selenium increase cellular proliferation long after reperfusion

**Fig. 7:**
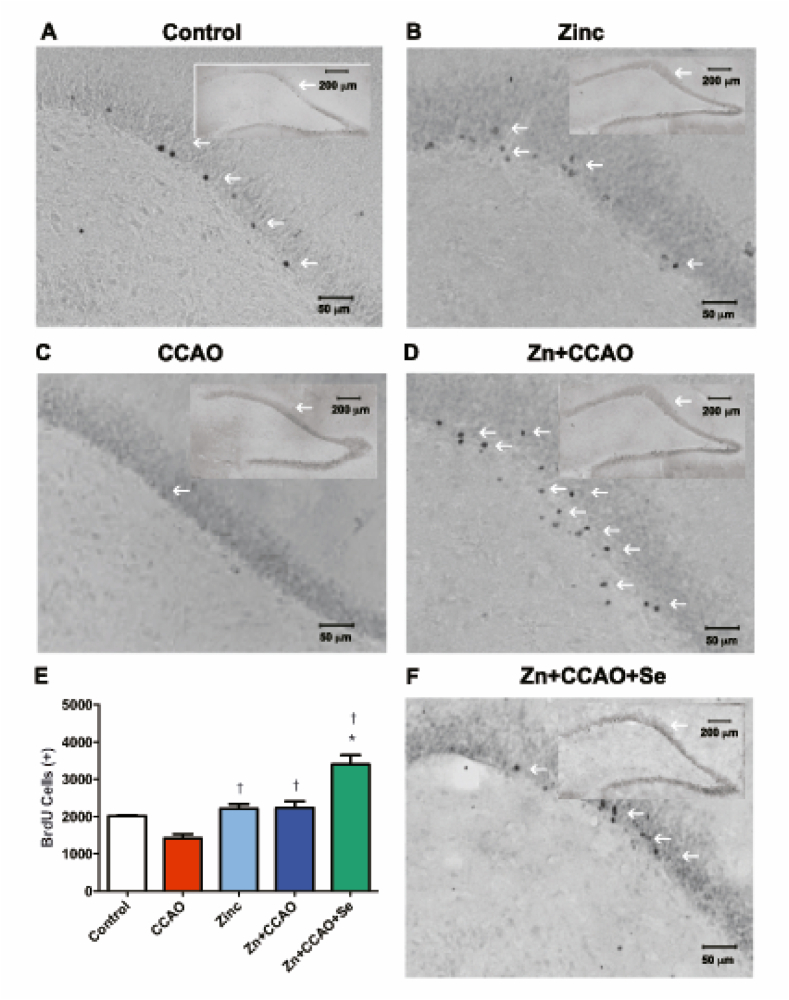
Prophylactic administration of zinc and therapeutic administration of selenium increased BrdU-positive cells in the dentate gyrus of the hippocampus. The representative micrographs presented in the upper right corner of each micrograph were integrated from several 20X amplifications; the large photo corresponds to a magnification of the area marked with an arrow in the integrated micrograph. The number of BrdU-positive cells is expressed on the graph. Values are the mean ± SEM of 24 slices of 3 independent brains obtained each 240 μm. Statistical differences are indicated as *P* < 0.05, as determined by the Two-way ANOVA test and Bonferroni's multiple comparison analysis, compared to *, the control group, or †, the CCAO group. The arrows indicate BrdU immunoreactivity.

**Fig. 8:**
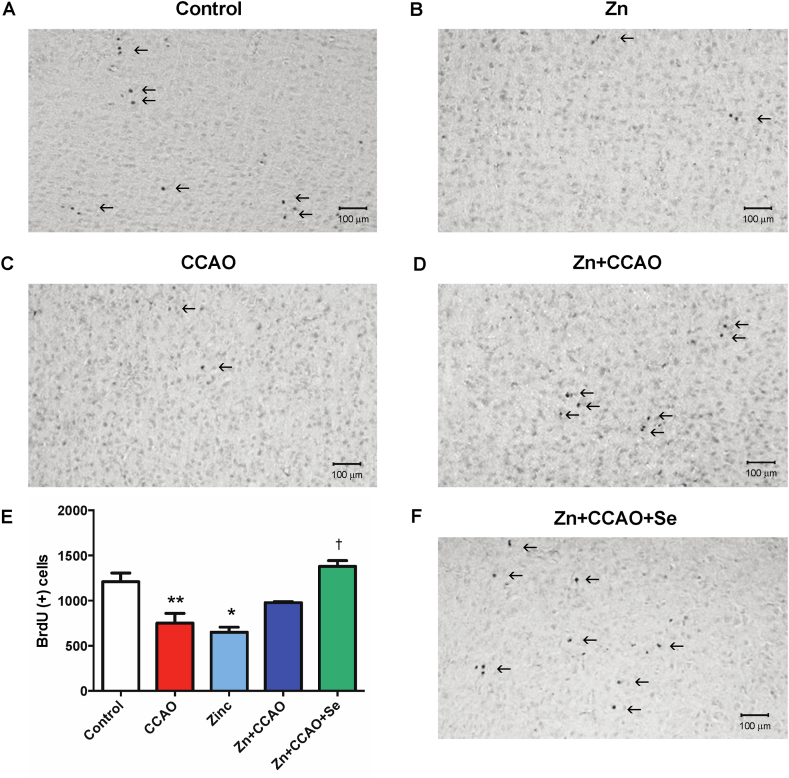
Prophylactic administration of zinc and therapeutic administration of selenium increased BrdU-positive cells in the temporoparietal cortex. The graph shows the number of BrdU-positive cells in the control and experimental groups. Values are the mean ± SEM of 24 slices of 3 independent brains obtained each 240 μm. Statistical differences are indicated as *P* < 0.05, as determined by the Two-way ANOVA test and Bonferroni's multiple comparison analysis when compared to either the * Control group or † the CCAO group. Arrows indicate BrdU immunoreactivity.

The hypoxia-ischemia (CCAO group) decreased the number of BrdU-positive cells in the temporoparietal cortex (37.9 ± 8.9 %, *P* < 0.05, Fig. 8C and E) compared with the group control (Fig. 8A and E). The Zn + CCAO + Se group showed an increase in the number of BrdU-positive cells in the hippocampus (133 % ± 37.9, *P* < 0.05, Fig. 7E and F) compared with the CCAO group (Fig. 7C and E) and in the temporoparietal cortex (90 % ± 16, *P* < 0.05, Fig. 8E and F) compared with the CCAO group (Fig. 8C and E). However, the number of BrdU-positive cells in the Zn and Zn + CCAO groups did not differ from that of the control group in the temporoparietal cortex (Fig. 8A, B, D, and E) compared with the control group and hippocampus (Fig. 7A, B, D, and E).

## 4 Discussion

Our results showed that the combined administration of prophylactic Zn and therapeutic Se in a 10-min transient CCAO rat model was more effective in reducing neurological sequelae than prophylactic Zn alone. The combined treatment significantly affected neuroinflammation by reducing reactive astrogliosis and lipid peroxidation; this effect was likely amplified by a significant increase in the expression of growth factors involved in neuronal repair during the early phase of transient ischemia [64]. The long-term cell proliferation observed in the treated groups may reflect the induction of a neuronal repair process. However, these beneficial effects were more consistently observed in the temporoparietal cortex than in the hippocampus.

Ischemic stroke is the fifth leading cause of death in people aged 15–59 years, and 15 % of all ischemic strokes occur in young adults and adolescents, according to the World Health Organization [13]. Depression and memory impairment are the most common late sequelae in adult human patients and experimental animal models. These effects have been shown to persist even after full recovery from a transient ischemic attack [5,6].

Transient hypoxia-ischemia in the CCAO group caused a differential regulation in growth factor mRNA transcription, the process is likely to be facilitated by the hypoxia-inducible factor (HIF) which is present in the early phase [[65], [66], [67]]. Likewise, the prophylactic administration of Zn alone or combined with therapeutic Se also increased growth factor expression, including NGF, BDNF, IGF-1, and FGF2, in the first 168 h post-reperfusion [33,68]. The effect of the growth factor expression on transient ischemic attack has been associated with preventing cognitive deficits and some behavior alterations due to a reduction in nitrosative stress and astrogliosis and increased cell proliferation, even 30 days post-reperfusion [[69], [70], [71]].

Previous studies have shown that prophylactic subacute Zn administration and swimming exercise [32] or chronic Zn and therapeutic Se administration have a neuroprotective effect at day 7 post-reperfusion [34]. The present work shows that chronic Zn administration combined with therapeutic Se can extend the neuroprotective effect for a long time post-reperfusion. The preconditioning effect of Zn could induce oxidative stress in the early phase after damage by stimulating response elements, it could promote the expression of growth factors and antioxidant enzymes, controlling neurological damage and inducing the regenerative phase. The therapeutic administration of Se would enhance this effect. However, therapeutic Se administration in the absence of prophylactic Zn supplementation increases lipid peroxidation, reactive astrogliosis, and decreased BrdU-positive cells at 30 days post-reperfusion (data not published), suggesting that the preconditioning stimulus in the early phase is necessary to promote long-term regeneration.

Zn supplementation has been demonstrated to regulate the expression of BDNF and its receptor [72] while also promoting pro-BDNF cleavage through matrix metalloproteinases [73,74]. These effects are associated with reduced ischemia-reperfusion injury in the spinal cord of rats [55]. The proposed mechanisms underpinning such an improvement are increased antiapoptotic activity and cell proliferation, synaptic plasticity, neurogenesis, and long-term Zn potentiation [55,[75], [76], [77]]. In this work, the combined Zn and Se administration improved memory consolidation for more than one month after reperfusion, which could be mediated by NGF mRNA upregulation, also observed here, as reported previously [78], suggesting the promotion of neurite outgrowth [41,79], differentiation, survival, and neuronal maturation in the brain [41]. Furthermore, NGF-mediated molecular mechanisms can induce the Zn finger protein 179 (Znf179) by an Sp1-dependent mechanism, which decreases oxidative stress to promote neuroprotection [80]. NGF also displays an anti-inflammatory effect [41] by reducing oxidative stress through GPx activity, a selenium-dependent enzyme [39], and restoring antioxidant enzymes such as catalase, GPx, and SOD [34]. In addition, the NGF effect on promoting neurite outgrowth can be potentiated by the antiapoptotic effect of Se [79] mediated by Bcl-2, which inhibits Bax protein expression [81], the release of the cytochrome *c*, and promotes the activation of caspase 3 and 9 [82].

Prophylactic chronic Zn administration increased IGF-1 and FGF2, as seen before, with subacute Zn administration [33]. The therapeutic effect of IGF-1 has been shown in several central nervous system insults, such as hypoglycemia-induced trauma, where IGF-1 increases glucose uptake in neurons through GLUT1 expression [83]. In addition, IGF-1 acts on neural plasticity [83,84] and myelin synthesis through the increase in oligodendrocyte proliferation, cell survival, and differentiation [85,86]. Previous studies have shown that in a mouse stroke model, IGF-1 released from microspheres induces the production of new neurons in the subventricular zone (SVZ), which then migrate toward the injured striatum [87]. Moreover, IGF-1 appears to be important in maintaining axonal and dendritic morphology. Promoting the growth of axons and dendrites, IGF-1 may improve the synaptic function [88].

BDNF and NGF have been proposed to modulate depression [49]. Therefore, it is plausible that BDNF and NGF, which were found to be upregulated here, could mediate the improved cognitive function and the reduced depression/anxiety-like behavior observed at long-term post-reperfusion by the combined Zn and Se administration or Zn administration alone.

After cerebral artery occlusion, a combination of prophylactic zinc and therapeutic selenium resulted in reduced anxiety and depression-like behaviors, as well as decreased levels of GFAP. However, the individual Zn administration increased GFAP expression after ischemia. The participation of astrocytes in anxiety and depression-like behavior is controversial since reactive astrocytes could be involved in scar formation and blood-brain barrier (BBB) impairments. Then, the reduction in astrocytosis could prevent scar formation and tissue damage [89]. Astrocytes are a type of brain cell that plays a role in synaptic plasticity, which can modify learning and memory. Specifically, they are involved in long-term potentiation (LTP) and long-term depression (LTD) [90]. Astrocytes are responsible for several cellular mechanisms related to learning and memory, including transmitter uptake, potassium buffering, and the release of gliotransmitters such as glutamate, ATP, and d-serine [91,92]. On this basis, Zn administration could improve learning and memory by increasing the number of reactive astrocytes.

The increase in growth factors may be responsible for inducing BrdU-positive cells, whether glia or neurons, from precursors that proliferated and matured on day 30 post-reperfusion. Accordingly, the combination of Zn and Se increased BrdU-positive cells. However, It decreased the GFAP-positive cells in the cerebral cortex and dentate gyrus. These findings suggest that cells that are positive for BrdU are newly generated neurons, rather than glial cells, which may contribute to the recovery of brain tissue after ischemic injury. This could account for the improvement of cognitive function.

Zn can prevent the increment of anxiety, corticosterone levels, IFN-γ, and GFAP-positive cells [93]. In addition, Zn has toxic effects on astrocytes, increasing ROS production and GSH thiol redox imbalance *in vitro* [94]. Protection of astrocytes through autocrine and paracrine mechanisms can be achieved by expressing ZnT-1 [95] and Cu–Zn SOD [96], Nrf2 activation, and heme oxygenase-1 induction [97]. It is plausible that those Zn neuroprotective mechanisms are involved in improving cognitive function and preventing anxiety/depression-like behavior. SELENBP1, SELENOP, and SEPS1 expression in astrocytes may regulate neural selenium levels [98]. These mechanisms could promote cell survival, antioxidant activity, and an anti-inflammatory effect, thus protecting astrocytes and neurons from an ischemic insult [99]. Induction of nuclear factors Nrf2, SOCS3/STAT3, and inhibition of apoptosis [100] may also be involved in the Zn and Se neuroprotective effect on memory recovery and prevention of anxiety/depression-like behavior on a long-term post-reperfusion.

Zn can act as an antidepressant drug [93], as shown by Zn supplementation and imipramine administration in a mouse model of depression [35]. In addition, Zn deficiency has been associated with major depression and higher Zn serum concentrations with a lower rate of symptoms related to depression and anxiety [101], due to deficits serotonergic system [102,103], BDNF, and neurogenesis [104,105]. Furthermore, Se deficiency has also been associated with depression, anxiety, cognitive decline, and suicidal behavior [106]. In a hyperglycemia model in mice, Se administration prevented anxiety-like behaviors [107] by modulating oxidative stress [108].

There are reports in focal cerebral ischemia models that preconditioning Zn promotes an antioxidant environment through the activation of copper/zinc SOD to favor proliferation, differentiation, and maturity of neuronal precursor cells through phosphorylation of PI3K, Akt and ERK [109,110], while Se through the PI3K, Akt-GSK3beta and Wnt pathway exerts a positive effect on the proliferation and survival of neural precursor cells [111]. In this work, the combined treatment of preconditioning Zn and therapeutic Se increased the number of BrdU-positive cells in the DG and temporoparietal cortex, suggesting cell proliferation of neuronal precursor cells. Therefore, the increment of BrdU-positive cells may be due to an improvement of the antioxidant environment through copper/zinc SOD and GPx4 enzymes [34,82].

In summary, the combined administration of prophylactic Zn and therapeutic Se produced a better long-term neuroprotective effect than Zn alone, inducing the upregulation of growth factors, decreasing lipid peroxidation and reactive astrogliosis, and improving neuronal survival. Those effects can add up to prevent memory loss and anxiety/depressive-like behavior, which are common chronic sequelae arising from transient ischemic attacks. Because these results are derived from animal models of cerebral vascular disease, further investigation is needed to determine their translational relevance to human neuroprotection. In addition, given the reported cytotoxic effects associated with high levels of Zn and Se, caution regarding dosage is imperative. Therefore, comprehensive exploration in animal models remains pivotal for elucidating the therapeutic window and optimizing the neuroprotective benefits of these minerals, which will determine their clinical potential in the prevention and management of cerebrovascular disorders.

## CRediT authorship contribution statement

**Constantino Tomas-Sanchez:** Validation, Methodology, Investigation. **Victor Manuel Blanco-Alvarez:** Methodology, Investigation. **Juan Antonio Gonzalez-Barrios:** Supervision, Methodology, Investigation, Funding acquisition, Formal analysis. **Daniel Martinez-Fong:** Writing – review & editing, Supervision. **Guadalupe Soto-Rodriguez:** Validation, Supervision, Methodology, Investigation. **Eduardo Brambila:** Methodology, Formal analysis. **Alejandro Gonzalez-Vazquez:** Methodology, Investigation, Formal analysis. **Ana Karina Aguilar-Peralta:** Methodology, Investigation. **Daniel I. Limón:** Methodology. **Viridiana Vargas-Castro:** Writing – original draft, Methodology, Investigation. **Jorge Cebada:** Supervision. **Victorino Alatriste-Bueno:** Methodology. **Bertha Alicia Leon-Chavez:** Writing – review & editing, Writing – original draft, Validation, Supervision, Methodology, Investigation.

## Declaration of competing interest

The authors declare the following financial interests/personal relationships which may be considered as potential competing interests:

Bertha Alicia Leon-Chavez reports financial support was provided by Autonomous University of Puebla. Juan Antonio Gonzalez Barrios reports financial support was provided by State Employees’ Social Security and Social Services Institute. If there are other authors, they declare that they have no known competing financial interests or personal relationships that could have appeared to influence the work reported in this paper.

## References

[1] Ekker M.S. (2018). Epidemiology, aetiology, and management of ischaemic stroke in young adults. Lancet Neurol..

[2] Panuganti K.K., Tadi P., Lui F. (2024). Treasure Island (F.L.) Ineligible Companies. Disclosure: Prasanna Tadi Declares No Relevant Financial Relationships with Ineligible Companies. Disclosure: Forshing Lui Declares No Relevant Financial Relationships with Ineligible Companies.

[3] Easton J.D. (2009). Definition and evaluation of transient ischemic attack: a scientific statement for healthcare professionals from the American heart association/American stroke association stroke Council; Council on Cardiovascular surgery and Anesthesia; Council on Cardiovascular Radiology and Intervention; Council on Cardiovascular Nursing; and the Interdisciplinary Council on peripheral vascular disease. The American Academy of Neurology affirms the value of this statement as an educational tool for neurologists. Stroke.

[4] Das J.K.R.G. (2018). Post stroke depression: the sequelae of cerebral stroke. Neurosci. Biobehav. Rev..

[5] Turner G.M. (2018). Establishing research priorities relating to the long-term impact of TIA and minor stroke through stakeholder-centred consensus. Res Involv Engagem.

[6] Medeiros G.C. (2020). Post-stroke depression: a 2020 updated review. Gen. Hosp. Psychiatr..

[7] Einstad M.S. (2021). Associations between post-stroke motor and cognitive function: a cross-sectional study. BMC Geriatr..

[8] Moran G.M. (2014). Fatigue, psychological and cognitive impairment following transient ischaemic attack and minor stroke: a systematic review. Eur. J. Neurol..

[9] Rafsten L., Danielsson A., Sunnerhagen K.S. (2018). Anxiety after stroke: a systematic review and meta-analysis. J. Rehabil. Med..

[10] Sagen-Vik U. (2022). The longitudinal course of anxiety, depression and apathy through two years after stroke. J. Psychosom. Res..

[11] Singhal A.B. (2013). Recognition and management of stroke in young adults and adolescents. Neurology.

[12] Boot E. (2020). Ischaemic stroke in young adults: a global perspective. J. Neurol. Neurosurg. Psychiatry.

[13] Namaganda P. (2022). Stroke in young adults, stroke types and risk factors: a case control study. BMC Neurol..

[14] Roy-O'Reilly M., McCullough L.D. (2018). Age and sex are critical factors in ischemic stroke pathology. Endocrinology.

[15] Duman R.S., Deyama S., Fogaca M.V. (2021). Role of BDNF in the pathophysiology and treatment of depression: activity-dependent effects distinguish rapid-acting antidepressants. Eur. J. Neurosci..

[16] Chen X.G. (2022). Longitudinal changes in the hypothalamic-pituitary-adrenal axis and sympathetic nervous system are related to the prognosis of stroke. Front. Neurol..

[17] Kim S. (2022). Dysregulated hypothalamic-pituitary-adrenal Axis is associated with increased inflammation and worse outcomes after ischemic stroke in diabetic mice. Front. Immunol..

[18] Chaturvedi P. (2020). Depression impedes neuroplasticity and quality of life after stroke. J. Fam. Med. Prim. Care.

[19] Passarelli J.P., Nimjee S.M., Townsend K.L. (2024). Stroke and neurogenesis: bridging clinical observations to new mechanistic insights from animal models. Transl Stroke Res.

[20] Tuo Q.Z. (2022). Thrombin induces ACSL4-dependent ferroptosis during cerebral ischemia/reperfusion. Signal Transduct. Targeted Ther..

[21] Allen L.A. (2020). Peri-ictal hypoxia is related to extent of regional brain volume loss accompanying generalized tonic-clonic seizures. Epilepsia.

[22] Nath K.A., Paller M.S. (1990). Dietary deficiency of antioxidants exacerbates ischemic injury in the rat kidney. Kidney Int..

[23] Adebayo O.L., Adenuga G.A., Sandhir R. (2016). Selenium and zinc protect brain mitochondrial antioxidants and electron transport chain enzymes following postnatal protein malnutrition. Life Sci..

[24] McCoy R.N. (1988). Oxidant stress following renal ischemia: changes in the glutathione redox ratio. Kidney Int..

[25] Omata Y. (2013). Decreased zinc availability affects glutathione metabolism in neuronal cells and in the developing brain. Toxicol. Sci..

[26] Bampi S.R. (2020). The selenocompound 1-methyl-3-(phenylselanyl)-1H-indole attenuates depression-like behavior, oxidative stress, and neuroinflammation in streptozotocin-treated mice. Brain Res. Bull..

[27] Taroza S. (2020). Deiodinases, organic anion transporter polypeptide polymorphisms and symptoms of anxiety and depression after ischemic stroke. J. Stroke Cerebrovasc. Dis..

[28] Wu Y. (2023). Selenoprotein gene mRNA expression evaluation during renal ischemia-reperfusion injury in rats and ebselen intervention effects. Biol. Trace Elem. Res..

[29] Wang J. (2018). Zinc, magnesium, selenium and depression: a review of the evidence, potential mechanisms and implications. Nutrients.

[30] Sajjadi S.S. (2022). The role of selenium in depression: a systematic review and meta-analysis of human observational and interventional studies. Sci. Rep..

[31] Yosaee S. (2022). Zinc in depression: from development to treatment: a comparative/dose response meta-analysis of observational studies and randomized controlled trials. Gen. Hosp. Psychiatr..

[32] Aguilar-Peralta A.K. (2022). Prophylactic zinc administration combined with swimming exercise prevents cognitive-emotional disturbances and tissue injury following a transient hypoxic-ischemic insult in the rat. Behav. Neurol..

[33] Blanco-Alvarez V.M. (2015). Prophylactic subacute administration of zinc increases CCL2, CCR2, FGF2, and IGF-1 expression and prevents the long-term memory loss in a rat model of cerebral hypoxia-ischemia. Neural Plast..

[34] Tomas-Sanchez C. (2018). Prophylactic zinc and therapeutic selenium administration increases the antioxidant enzyme activity in the rat temporoparietal cortex and improves memory after a transient hypoxia-ischemia. Oxid. Med. Cell. Longev..

[35] Wrobel A. (2015). The effect of imipramine, ketamine, and zinc in the mouse model of depression. Metab. Brain Dis..

[36] Ding Q. (2016). Zinc and imipramine reverse the depression-like behavior in mice induced by chronic restraint stress. J. Affect. Disord..

[37] Rafalo-Ulinska A. (2020). Imipramine influences body distribution of supplemental zinc which may enhance antidepressant action. Nutrients.

[38] Alim I. (2019). Selenium drives a transcriptional adaptive program to block ferroptosis and treat stroke. Cell.

[39] Shi Y. (2022). Selenium alleviates cerebral ischemia/reperfusion injury by regulating oxidative stress, mitochondrial fusion and ferroptosis. Neurochem. Res..

[40] Shultz S.R. (2015). Sodium selenate reduces hyperphosphorylated tau and improves outcomes after traumatic brain injury. Brain.

[41] Ozbal S. (2008). The effects of selenium against cerebral ischemia-reperfusion injury in rats. Neurosci. Lett..

[42] Colucci-D'Amato L., Speranza L., Volpicelli F. (2020). Neurotrophic factor BDNF, physiological functions and therapeutic potential in depression, neurodegeneration and brain cancer. Int. J. Mol. Sci..

[43] Hassan T.M., Yarube I.U. (2018). Peripheral brain-derived neurotrophic factor is reduced in stroke survivors with cognitive impairment. Pathophysiology.

[44] Poon C.H., Heng B.C., Lim L.W. (2021). New insights on brain-derived neurotrophic factor epigenetics: from depression to memory extinction. Ann. N. Y. Acad. Sci..

[45] Tang M.M. (2018). Fibroblast growth factor 2 modulates hippocampal microglia activation in a neuroinflammation induced model of depression. Front. Cell. Neurosci..

[46] Bryant E.M., Richardson R., Graham B.M. (2022). The association between salivary FGF2 and physiological and psychological components of the human stress response. Chronic Stress (Thousand Oaks).

[47] Cao J.Y. (2018). Expression of nerve growth factor carried by pseudotyped lentivirus improves neuron survival and cognitive functional recovery of post-ischemia in rats. CNS Neurosci. Ther..

[48] Aloe L. (2016). Nerve growth factor: role in growth, differentiation and controlling cancer cell development. J. Exp. Clin. Cancer Res..

[49] Mondal A.C., Fatima M. (2019). Direct and indirect evidences of BDNF and NGF as key modulators in depression: role of antidepressants treatment. Int. J. Neurosci..

[50] Arinami H. (2023). Serum cortisol and insulin-like growth factor 1 levels in major depressive disorder and schizophrenia. Sci. Rep..

[51] Fasil D.M. (2021). Selenium and zinc oxide multinutrient supplementation enhanced growth performance in zebra fish by modulating oxidative stress and growth-related gene expression. Front. Bioeng. Biotechnol..

[52] Ding J., Zhang Y. (2022). Relationship between the circulating selenium level and stroke: a meta-analysis of observational studies. J. Am. Nutraceutical Assoc..

[53] Zhuo Z. (2023). Selenium supplementation provides potent neuroprotection following cerebral ischemia in mice. J. Cerebr. Blood Flow Metabol..

[54] Wang W.M. (2015). The zinc ion chelating agent TPEN attenuates neuronal death/apoptosis caused by hypoxia/ischemia via mediating the pathophysiological cascade including excitotoxicity, oxidative stress, and inflammation. CNS Neurosci. Ther..

[55] Wang Y. (2014). Supplement zinc as an effective treatment for spinal cord ischemia/reperfusion injury in rats. Brain Res..

[56] Sowa-Kucma M. (2011). Chronic treatment with zinc and antidepressants induces enhancement of presynaptic/extracellular zinc concentration in the rat prefrontal cortex. Amino Acids.

[57] (2011). Guide for the Care and Use of Laboratory Animals.

[58] Morris R. (1984). Developments of a water-maze procedure for studying spatial learning in the rat. J. Neurosci. Methods.

[59] Seibenhener M.L., Wooten M.C. (2015). Use of the Open Field Maze to measure locomotor and anxiety-like behavior in mice. J. Vis. Exp..

[60] Kraeuter A.K., Guest P.C., Sarnyai Z. (2019). The elevated plus maze test for measuring anxiety-like behavior in rodents. Methods Mol. Biol..

[61] Gonzalez-Barrios J.A. (2002). Nitric oxide and nitric oxide synthases in the fetal cerebral cortex of rats following transient uteroplacental ischemia. Brain Res..

[62] Gerard-Monnier D. (1998). Reactions of 1-methyl-2-phenylindole with malondialdehyde and 4-hydroxyalkenals. Analytical applications to a colorimetric assay of lipid peroxidation. Chem. Res. Toxicol..

[63] Vargas-Castro V. (2021). Long-term taurine administration improves motor skills in a tubulinopathy rat model by decreasing oxidative stress and promoting myelination. Mol. Cell. Neurosci..

[64] Lu Y. (2020). Neuron-derived estrogen is critical for astrocyte activation and neuroprotection of the ischemic brain. J. Neurosci..

[65] Liu Q. (2017). Insulin-like growth factor 1 receptor-mediated cell survival in hypoxia depends on the promotion of autophagy via suppression of the PI3K/Akt/mTOR signaling pathway. Mol. Med. Rep..

[66] Turovskaya M.V. (2020). BDNF overexpression enhances the preconditioning effect of brief episodes of hypoxia, promoting survival of GABAergic neurons. Neurosci. Bull..

[67] Wu Q. (2021). Nerve growth factor (NGF) with hypoxia response elements loaded by adeno-associated virus (AAV) combined with neural stem cells improve the spinal cord injury recovery. Cell Death Dis..

[68] Li Y. (2022). Zinc improves neurological recovery by promoting angiogenesis via the astrocyte-mediated HIF-1alpha/VEGF signaling pathway in experimental stroke. CNS Neurosci. Ther..

[69] Behl T., Kotwani A. (2017). Downregulated brain-derived neurotrophic factor-induced oxidative stress in the pathophysiology of diabetic retinopathy. Can. J. Diabetes.

[70] Won L., Kraig R.P. (2021). Insulin-like growth factor-1 inhibits nitroglycerin-induced trigeminal activation of oxidative stress, calcitonin gene-related peptide and c-Fos expression. Neurosci. Lett..

[71] Zhang Z., Sun G.Y., Ding S. (2021). Glial cell line-derived neurotrophic factor and focal ischemic stroke. Neurochem. Res..

[72] Sowa-Kucma M. (2008). Antidepressant-like activity of zinc: further behavioral and molecular evidence. J. Neural. Transm..

[73] Hwang J.J. (2005). Activation of the Trk signaling pathway by extracellular zinc. Role of metalloproteinases. J. Biol. Chem..

[74] Hwang J.J., Park M.H., Koh J.Y. (2007). Copper activates TrkB in cortical neurons in a metalloproteinase-dependent manner. J. Neurosci. Res..

[75] Bekinschtein P. (2008). BDNF is essential to promote persistence of long-term memory storage. Proc. Natl. Acad. Sci. U. S. A..

[76] Bambah-Mukku D. (2014). A positive autoregulatory BDNF feedback loop via C/EBPbeta mediates hippocampal memory consolidation. J. Neurosci..

[77] Kowianski P. (2018). BDNF: a key factor with multipotent impact on brain signaling and synaptic plasticity. Cell. Mol. Neurobiol..

[78] Kheirvari S. (2006). Increased nerve growth factor by zinc supplementation with concurrent vitamin A deficiency does not improve memory performance in mice. J. Nutr. Sci. Vitaminol..

[79] Dang Z. (2019). Nerve growth factor gene therapy improves bone marrow sensory innervation and nociceptor-mediated stem cell release in a mouse model of type 1 diabetes with limb ischaemia. Diabetologia.

[80] Chuang J.Y. (2017). Specificity protein 1-zinc finger protein 179 pathway is involved in the attenuation of oxidative stress following brain injury. Redox Biol..

[81] Tan Y.X. (2007). [Effects of nerve growth factor pretreatment on apoptosis of neurons and expression of Bcl-2, Bax protein in brain tissue following cerebral ischemia/reperfusion injury in gerbils]. Zhongguo Wei Zhong Bing Ji Jiu Yi Xue.

[82] Yeo J.E., Kang S.K. (2007). Selenium effectively inhibits ROS-mediated apoptotic neural precursor cell death in vitro and in vivo in traumatic brain injury. Biochim. Biophys. Acta.

[83] Verleysdonk S. (2004). Regulation by insulin and insulin-like growth factor of 2-deoxyglucose uptake in primary ependymal cell cultures. Neurochem. Res..

[84] Anson K.J., Corbet G.A., Palmer A.E. (2021). Zn(2+) influx activates ERK and Akt signaling pathways. Proc. Natl. Acad. Sci. U. S. A..

[85] Bibollet-Bahena O., Cui Q.L., Almazan G. (2009). The insulin-like growth factor-1 axis and its potential as a therapeutic target in central nervous system (CNS) disorders. Cent. Nerv. Syst. Agents Med. Chem..

[86] Huang E. (2012). Growth hormone synergizes with BMP9 in osteogenic differentiation by activating the JAK/STAT/IGF1 pathway in murine multilineage cells. J. Bone Miner. Res..

[87] Nakaguchi K. (2012). Growth factors released from gelatin hydrogel microspheres increase new neurons in the adult mouse brain. Stem Cell. Int..

[88] Madathil S.K., Saatman K.E., Kobeissy F.H. (2015). Brain Neurotrauma: Molecular, Neuropsychological, and Rehabilitation Aspects.

[89] Balkaya M. (2021). CD36 deficiency reduces chronic BBB dysfunction and scar formation and improves activity, hedonic and memory deficits in ischemic stroke. J. Cerebr. Blood Flow Metabol..

[90] Durkee C. (2021). Astrocyte and neuron cooperation in long-term depression. Trends Neurosci..

[91] Papouin T. (2017). Astrocytic control of synaptic function. Philos. Trans. R. Soc. Lond. B Biol. Sci..

[92] Durkee C.A., Araque A. (2019). Diversity and specificity of astrocyte-neuron communication. Neuroscience.

[93] Kirsten T.B. (2020). zinc, but not paracetamol, prevents depressive-like behavior and sickness behavior, and inhibits interferon-gamma and astrogliosis in rats. Brain Behav. Immun..

[94] Bishop G.M., Dringen R., Robinson S.R. (2007). Zinc stimulates the production of toxic reactive oxygen species (ROS) and inhibits glutathione reductase in astrocytes. Free Radic. Biol. Med..

[95] Nolte C. (2004). ZnT-1 expression in astroglial cells protects against zinc toxicity and slows the accumulation of intracellular zinc. Glia.

[96] Wang J., Ma J.H., Giffard R.G. (2005). Overexpression of copper/zinc superoxide dismutase decreases ischemia-like astrocyte injury. Free Radic. Biol. Med..

[97] Shin J.H. (2012). Ethyl pyruvate-mediated Nrf2 activation and hemeoxygenase 1 induction in astrocytes confer protective effects via autocrine and paracrine mechanisms. Neurochem. Int..

[98] Sasuclark A.R., Khadka V.S., Pitts M.W. (2019). Cell-type specific analysis of selenium-related genes in brain. Antioxidants.

[99] Fradejas N. (2008). SEPS1 gene is activated during astrocyte ischemia and shows prominent antiapoptotic effects. J. Mol. Neurosci..

[100] Turovsky E.A. (2022). Features of the cytoprotective effect of selenium nanoparticles on primary cortical neurons and astrocytes during oxygen-glucose deprivation and reoxygenation. Sci. Rep..

[101] Anbari-Nogyni Z. (2020). Relationship of zinc status with depression and anxiety among elderly population. Clin Nutr ESPEN.

[102] Doboszewska U. (2017). Zinc in the monoaminergic theory of depression: its relationship to neural plasticity. Neural Plast..

[103] Portbury S.D., Adlard P.A. (2017). Zinc signal in brain diseases. Int. J. Mol. Sci..

[104] Gower-Winter S.D., Levenson C.W. (2012). Zinc in the central nervous system: from molecules to behavior. Biofactors.

[105] Camuso S. (2022). Pleiotropic effects of BDNF on the cerebellum and hippocampus: implications for neurodevelopmental disorders. Neurobiol. Dis..

[106] Sher L. (2008). Depression and suicidal behavior in alcohol abusing adolescents: possible role of selenium deficiency. Minerva Pediatr..

[107] Dos Santos M.M. (2018). Hyperglycemia elicits anxiety-like behaviors in zebrafish: protective role of dietary diphenyl diselenide. Prog. Neuro-Psychopharmacol. Biol. Psychiatry.

[108] Bampi S.R. (2019). Repeated administration of a selenium-containing indolyl compound attenuates behavioural alterations by streptozotocin through modulation of oxidative stress in mice. Pharmacol. Biochem. Behav..

[109] Ohashi K. (2015). Zinc promotes proliferation and activation of myogenic cells via the PI3K/Akt and ERK signaling cascade. Exp. Cell Res..

[110] Takemura S. (2006). Correlation between copper/zinc superoxide dismutase and the proliferation of neural stem cells in aging and following focal cerebral ischemia. J. Neurosurg..

[111] Zheng R. (2017). Selenomethionine promoted hippocampal neurogenesis via the PI3K-Akt-GSK3beta-Wnt pathway in a mouse model of Alzheimer's disease. Biochem. Biophys. Res. Commun..

